# Combination of Amphiphilic Cyclic Peptide [R_4_W_4_] and Levofloxacin against Multidrug-Resistant Bacteria

**DOI:** 10.3390/antibiotics11030416

**Published:** 2022-03-20

**Authors:** Muhammad Imran Sajid, Sandeep Lohan, Shun Kato, Rakesh Kumar Tiwari

**Affiliations:** 1 Center for Targeted Drug Delivery, Department of Biomedical and Pharmaceutical Sciences, Chapman University School of Pharmacy, Harry and Diane Rinker Health Science Campus, Irvine, CA 92618, USA; sajid@chapman.edu (M.I.S.); lohan@chapman.edu (S.L.); skato@chapman.edu (S.K.); 2 Faculty of Pharmacy, University of Central Punjab, Lahore 54000, Pakistan

**Keywords:** antibacterial resistance, cyclic antimicrobial peptides, checkerboard assay, drug-resistant bacteria, combination therapy

## Abstract

Bacterial resistance is a growing global concern necessitating the discovery and development of antibiotics effective against the drug-resistant bacterial strain. Previously, we reported a cyclic antimicrobial peptide [R_4_W_4_] containing arginine (R) and tryptophan (W) with a MIC of 2.67 µg/mL (1.95 µM) against methicillin-resistant *Staphylococcus aureus* (MRSA). Herein, we investigated the cyclic peptides [R_4_W_4_] or linear (R_4_W_4_) and their conjugates (covalent or noncovalent) with levofloxacin (Levo) with the intent to improve their potency to target drug-resistant bacteria. The physical mixture of the Levo with the cyclic [R_4_W_4_] proved to be significantly effective against all strains of bacteria used in the study as compared to covalent conjugation. Furthermore, the checkerboard assay revealed the significant synergistic effect of the peptides against all studied strains except for the wild type *S. aureus*, in which the partial synergy was observed. The hemolysis assay revealed less cytotoxicity of the physical mixture of the Levo with [R_4_W_4_] (22%) as compared to [R_4_W_4_] alone (80%). The linear peptide (R_4_W_4_) and the cyclic [R_4_W_4_] demonstrated ~90% and 85% cell viability at 300 µg/mL in the triple-negative breast cancer cells (MDA-MB-231) and the normal kidney cells (HEK-293), respectively. Similar trends were also observed in the cell viability of Levo-conjugates on these cell lines. Furthermore, the time-kill kinetic study of the combination of [R_4_W_4_] and Levo demonstrate rapid killing action at 4 h for MRSA (ATCC BAA-1556) and 12 h for *E. coli* (ATCC BAA-2452), *P. aeruginosa* (ATCC BAA-1744), and *K. pneumoniae* (ATCC BAA-1705). These results provide the effectiveness of a combination of Levo with cyclic [R_4_W_4_] peptide, which may provide an opportunity to solve the intriguing puzzle of treating bacterial resistance.

## 1. Introduction

Antimicrobial resistance (AMR) is a growing global concern that has caused prolonged treatment for many common infections [1]. Overuse of antibiotics for the treatment of bacterial infections has promoted the development of antibacterial resistance genes, rendering many antimicrobial treatments ineffective after a short period [2]. Methicillin-resistant *Staphylococcus aureus* (MRSA) is a Gram-positive bacteria showing increased antibiotic resistance. MRSA poses an enormous threat to the medical field, causing over 60% of Staphylococcal infections in the intensive care unit [3]. In addition, the antibiotics developed to combat the diseases caused by drug-resistant bacteria, such as daptomycin, have already shown resistance to MRSA strains [4]. Besides, the emergence of different strains of bacteria having varying susceptibility to existing antibiotics challenges the use of a single antibiotic for the recalcitrant bacteria, necessitating a combination of antibiotics showing synergistic activity [5,6,7,8,9]. 

As antimicrobial resistance increases, there is a prominent need for other means of treatment; a promising area of success is antimicrobial peptides (AMPs) [3,10]. Antimicrobial peptides were first discovered in the 1980s from natural sources comprising sequences containing 4 to 50 amino acid residues chiefly composed of cationic and hydrophobic residues [11,12]. AMPs utilize their amphipathic structure to target a bacteria’s lipopolysaccharide layer, binding to the lipid components and the phospholipid groups of a bacteria’s cell membrane. This mechanism causes a bacteria’s cell membrane lysis, rendering it immobile [5,13,14]. Although much scientific literature points to their tremendous potential, only a few limited peptide-based antibiotics have been granted regulatory approval. Several reasons for this limited bench to bedside translation are attributed due to their associated toxicity to mammalian cells [15] and low stability [16,17,18]. Synthetic antimicrobial peptides (SAMPs) offer several advantages to conventional AMPs, such as low toxicity, cost-effectiveness, and highly potent antibacterial activity [19,20]. Several research groups, including ours, seek to investigate the usefulness of synthetic antimicrobial peptides to combat this global health threat [21,22].

Previously, our lab reported a cyclic peptide containing four tryptophan (W) and arginine (R) amino acids [R_4_W_4_] as a synthetic antimicrobial peptide showing potent antibacterial activity against clinically resistant bacterial strains [23]. In particular, it showed a minimum inhibitory concentration (MIC) of 2.67 µg/mL (1.95 µM) against MRSA (ATCC 43300) and 42.8 µg/mL (31.3 µM) against *P. aeruginosa* (PAO1) [24]. In addition, the time-kill studies revealed that [R_4_W_4_] and tetracycline combination exhibited bactericidal activity against MRSA and *E. coli* (ATCC 35218). Furthermore, we synthesized several analogs of [R_4_W_4_] to obtain a structure-antibacterial activity. We deduced that the addition of more than nine amino acids or lower than six amino acids in a cyclic ring resulted in low antibacterial potency compared to [R_4_W_4_]. It was concluded that [R_4_W_4_], [R_3_W_4_], and [R_4_W_3_] peptides showed similar potency with a MIC value of ~2.97 µg/mL (~2.1 µM) [24]. Furthermore, a combination of [R_4_W_4_] alone and in combination with current first-line antibiotics (isoniazid or pyrazinamide) showed efficacious in treating *Mycobacterium tuberculosis* (M. tb) inside human granulomas [25].

Antibiotic-peptide conjugates or antibiotic hybrids have been designed, evaluated, and found to be more efficacious to treat pathogenic bacteria compared to the individual antibiotics or peptides [26,27]. However, conjugates were also reported with lower potency compared to antibiotics due to the covalent linkage between them and their incomplete hydrolysis to afford interaction with the drug target. For example, [R_4_W_4_] was conjugated through an amide bond using lysine as a linker with the levofloxacin (Levo) to synthesize Levo-[W_4_R_4_K] conjugate to generate enhanced potency against MRSA and other Gram-negative bacteria. However, conjugation resulted in a decrease in antibacterial activity. For instance, [R_4_W_4_K]-levofloxacin-Q conjugate showed a MIC value of 32 µg/mL against MRSA and >128 µg/mL against *Klebsiella pneumonia*, whereas the physical mixture of [R_4_W_4_] and Levo showed a MIC of 8 µg/mL against MRSA and 32 µg/mL against *Klebsiella pneumonia* [28,29,30]. These outcomes suggested the stability of conjugate, which impacted their individual activity as suggested by the physical mixture of Levo and [R_4_W_4_]. In a different study, Levo/Indolicidin was conjugated with a transporter peptide using an amide and ester linkage, concluding that the conjugate containing amide linkage presented less activity compared to the ester linkage [31]. In these studies, the carboxylic functionality of Levo, which is the key structural element responsible for antibacterial activity, was exploited to synthesize antibiotic peptide conjugate through amide bond [23,31]. The amide linkage’s overall stability, especially under in-vitro experimental conditions, could have inhibited the release of Levo, and ultimately, favorable interaction with the target pathogen resulted in a decrease in the potency of the Levo-[R_4_W_4_] conjugate. Therefore, we hypothesized to synthesize the Levo-[R_4_W_4_] conjugate using an ester linkage, which is prone to undergo hydrolysis in physiological conditions to release peptide and antibiotics to exert a synergistic activity. Given the potency and broad-spectrum antibacterial activity of individual Levo and peptides against MRSA, there is a strong probability of effective synergistic action by complementing each other’s unique mode of action to provide compounds against multi-drug resistant strain of MRSA. Several studies demonstrated the synergism of AMPs and conventional antibiotics [32,33]. In addition, we envisioned that the multifaceted mode of action of the conjugate poses a challenging task for the target pathogen to suppress or subvert antibacterial action and evolve to be immune to the treatment. 

In this report, the antibacterial activity of combination (covalent or physical mixture) or individual compound (Levo and peptides) was determined against a panel of eight bacterial strains by including one resistant strain of each type of bacteria. We sought to determine the synergistic activity of the physical mixture using a checkerboard assay. The cytotoxicity profile of all test compounds (Levo and peptide alone, chemical conjugates, and physical combination) was assessed by measuring the lytic activity against human red blood cells. Finally, we determined the time-kill kinetics of the studied antibacterial compounds. These studies will provide a better understanding of using a combination of antibiotics and antibacterial peptides to treat infections caused by multi-drug resistant bacteria.

## 2. Results and Discussion

### 2.1. Synthesis and Purification of the Compounds

**Scheme 1:**
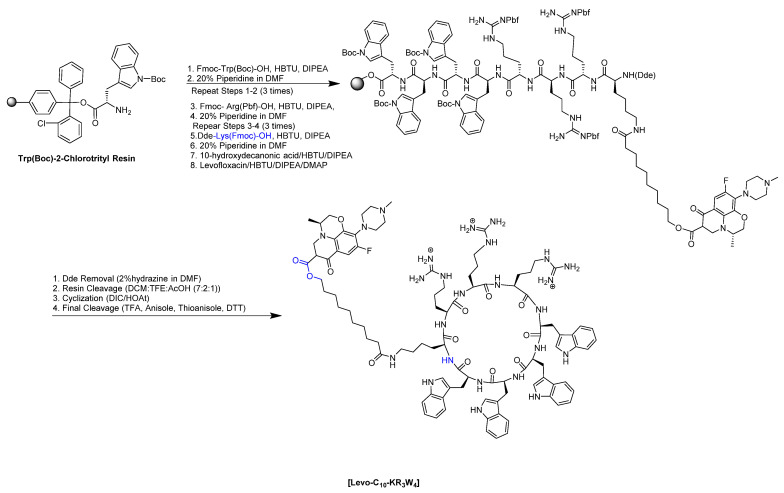
Synthesis of cyclic peptide-Levo conjugate [Levo-C_10_-KR_3_W_4_].

**Figure 1:**
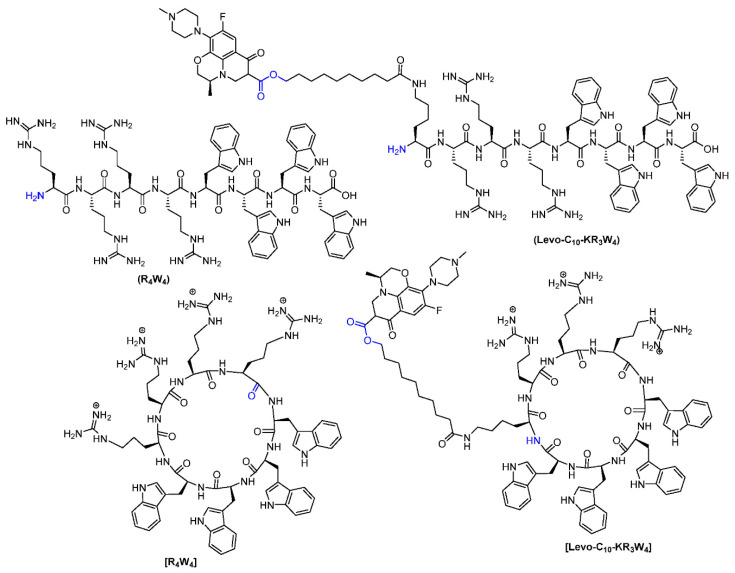
Chemical structures of synthesized peptides and conjugates.

The synthesis of linear (R_4_W_4_) and cyclic [R_4_W_4_] peptides were performed using Fmoc/tBu solid-phase peptide chemistry as reported earlier. Scheme 1 depicts the synthesis of cyclic peptides conjugated with Levo. The design of synthesis of Levo conjugates with antibacterial peptide was achieved by substituting one arginine residue with lysine to afford to couple a linker, 10-hydroxy decanoic acid (10-HDA), to the side chain of lysine. This linker contains 10 carbons (C_10_) to provide sufficient spacer from antibacterial peptide side-chain residues to avoid steric hindrance to assist in the conjugation of Levo and contribute to overall hydrophobicity. Due to the use of an unprotected OH group in 10-HDA during coupling to peptidyl resin using 2-(1H-benzotriazol-1-yl)-1,1,3,3-tetramethyluronium hexafluorophosphate (HBTU) and N, N-diisopropylethylamine (DIPEA), a urea (uronium) adduct at the OH group of conjugated decanoic acid was formed with the peptide, which reduced the availability of the OH group for further conjugation with Levo. It was confirmed due to an increase of mass with ~99 dalton in the MALDI mass spectrum for the formation of uronium adduct in the peptide (Supplementary Information). An ester linkage was generated between the OH group of the linker and COOH of Levo using HBTU, DIPEA, and 4-dimethylaminopyridine (DMAP) as coupling and activating reagents (Scheme 1). Ester linkage was susceptible to esterase under physiological conditions for faster hydrolysis and release of drug from conjugates [34]. Figure 1 shows the chemical structures of all the peptides and conjugates used in these studies. All the peptides and conjugates were fully characterized using matrix-assisted laser desorption/ionization time-of-flight (MALDI-TOF) mass spectrometry and purified using reverse-phase high-performance liquid chromatography (RP-HPLC) and were >95% pure as analyzed by analytical HPLC (Supplementary Information). The Supplementary Material provides MALDI-TOF characterization data of peptides and conjugates.

### 2.2. Antibacterial Activity

The antibacterial activity of synthesized peptides, conjugates, and their physical mixture are reported in Table 1. Levo and cyclic peptide [R_4_W_4_] showed a similar efficacy against *S. aureus* (ATCC 29213) and MRSA (ATCC BAA-1556) with a MIC of 3.13 µg/mL (2.28 µM), which is consistent with reported MICs [23]. The linear peptide (R_4_W_4_) was two-fold less active against both Gram-positive strains with a MIC of 6.25 µg/mL (4.51 µM). Cyclic peptide [R_4_W_4_] exhibited good activity (MIC = 6.25–12.5 µg/mL) against all the tested strains of *E. coli* and *K. pneumonia*. However, Levo displayed mild activity (MIC = 25–50 µg/mL) (69.18–138.36 µM) against *E. coli* and *K. pneumonia*. Similar to previous reports, Levo was highly active against both the strains of *P. aeruginosa* with a MIC of 0.78 µg/mL (2.16 µM) [30]. [R_4_W_4_] showed similar activity against both strains of *P. aeruginosa* with a MIC of 12.5 µg/mL (9.11 µM). R_4_W_4_ was found to be moderately active against all tested strains of Gram-negative bacteria with a MIC of 25–50 µg/mL (18.03–36.06 µM). Interestingly, contrary to peptide alone, the linear peptide (R_4_W_4_) conjugate with Levo, (Levo-C_10_-KR_3_W_4_) showed two-fold higher activity against *S. aureus* and MRSA (MIC = 12.5 µg/mL (6.67 µM)) as compared to cyclic peptide Levo conjugate [Levo-C_10_-KR_3_W_4_] (MIC = 25–50 µg/mL (13.45–26.90 µM)). A similar conjugate of these antibacterial peptides containing Levo with amide linkages; (R_4_W_4_K-Levo) and [R_4_W_4_K-Levo] demonstrated higher MICs 32–128 µg/mL (~16.75–66.35 µM) against MRSA and *K. pneumoniae* [30]. The use of ester linkage slightly improved potency in these newly synthesized conjugates during the in-vitro assay. Very interesting, the ester bond conjugates showed one to two-fold higher potency as compared to amide bond conjugate. In addition, in some instances, it was found that linear peptide ester conjugate showed one-fold higher potency as compared to cyclic peptide conjugates, e.g., *S. aureus*, MRSA, and *K. pneumoniae* (ATCC 13883), which may be due to easier hydrolysis of conjugate to release linear peptide and Levo as compared to hydrolysis of cyclic constrained peptide conjugate. Furthermore, both the covalent conjugates of Levo with peptides showed mild to moderate activity (MIC = 25–100 µg/mL (~13.34–53.79 µM)) against all tested Gram-negative strains. The conjugates may be more effective during in vivo studies where the ester linkage will release both the peptide and Levo for antibacterial activity. In the conjugates, the COOH of Levo has been used in the ester linkage, which is part of the pharmacophore for antibacterial activity.

The addition of bulky peptides with the COOH group of Levo provides further steric hindrance. Furthermore, the conjugates also provided specific interaction with bacterial membrane for membranolytic activity as compared to peptide alone. Therefore, these conjugates did not show enhanced potency as compared to Levo or peptide alone.

Nonetheless, a close examination of antibacterial activity in Table 1 revealed that the physical mixture of Levo with either linear or cyclic peptides (R_4_W_4_ or [R_4_W_4_]) exerted high antibacterial activity against all tested strains as compared to Levo and peptides alone and their covalent conjugates. The physical mixture was performed at the 1:1 *w*/*w* ratio that contains 3:85:1 molar ratio of Levo and peptides. The physical mixture of Levo and linear peptide (R_4_W_4_) exhibited good activity with a MIC of 3.13 µg/mL against both *S. aureus* and MRSA. However, for the physical mixture of Levo and cyclic peptide [R_4_W_4_], a similar trend of activity against *S. aureus* (MIC = 3.13 µg/mL) was observed, but there was a one-fold increase in MIC (6.25 µg/mL) against MRSA (Table 1). Noticeably, physical mixture of Levo and peptides exerted the killing action against both strains of *E. coli* at a 2 to 4-fold lesser MICs (12.5–25 µg/mL) as compared to Levo (MIC = 50 µg/mL) but 2-fold higher MICs (6.25–12.50 µg/mL) against peptides alone. For *P. aeruginosa*, while the physical mixture of Levo and peptides displayed activity at one-fold higher MICs (0.78–1.56 µg/mL) as compared to Levo (0.78 µg/mL) alone, a significant decrease in MIC was observed as compared to peptides (12.5–25 µg/mL) alone and their covalent conjugates with Levo (≥100 µg/mL). Similarly, compared to Levo alone (MIC = 25–50 µg/mL), the physical mixture exhibited around four-fold decrease in MICs (12.5–25 µg/mL) against imipenem-resistant *K. pneumonia*. Therefore, it was concluded that the physical combination is more potent than the chemical conjugates of Levo and antibacterial peptides containing either an amide bond or an ester linkage due to higher molar ratio of Levo. Considering the exciting outcomes of the physical mixture, we further investigate the synergistic activity of Levo and peptides described below.

### 2.3. Combination Studies Using Levo and Peptides

Since the physical mixture exhibited a significantly higher potency against almost all bacterial strains used in the study as compared to covalent conjugates, therefore, a checkerboard assay was performed to examine if the combination of antibacterial peptides with Levo has a synergistic or additive effect on antibacterial activity. Both the susceptible and antibiotic-resistant strains of bacteria were used in this study. Table 2 revealed the antibacterial activity of the combination of Levo and peptides (linear or cyclic). As the data suggest, linear peptide (R_4_W_4_) showed partial synergy with Levo for *S. aureus* (FICI = 0.748) and MRSA (FICI = 0.999). However, the combination of Levo and cyclic peptide [R_4_W_4_] displayed complete synergism (FICI = 0.498) against MRSA. In contrast, the combination of Levo and linear peptide (R_4_W_4_) was found to be less effective against Gram-negative strains with a non-significant synergism was observed for *E. coli* (ATCC 25922) (FICI = 1), *P. aeruginosa* (ATCC 27883) (FICI = 1.062), and *K. pneumoniae* (ATCC 13883) (FICI = 1.25). However, a partial synergistic activity (FICI = 0.562) was observed against carbapenem-resistant *P. aeruginosa* (ATCC BAA-1744).

Interestingly, the combination of Levo and cyclic peptide [R_4_W_4_] showed synergy against all the studied bacterial strains except for *S. aureus* (ATCC 29213), which exhibited partial synergy (FICI = 0.748) (Table 2). The results of the synergistic study explicitly revealed the effectiveness of the physical mixture over Levo and peptide alone and their chemical conjugates. This observation can be further investigated for other potent membranolytic antibiotics such as daptomycin, polymyxin, telavancin, oritavancin, and dalbavancin [5]. Further exploration of these studies can result in lead combinations that can be translated for in-vivo studies in the future.

### 2.4. Hemolytic Study

**Figure 2:**
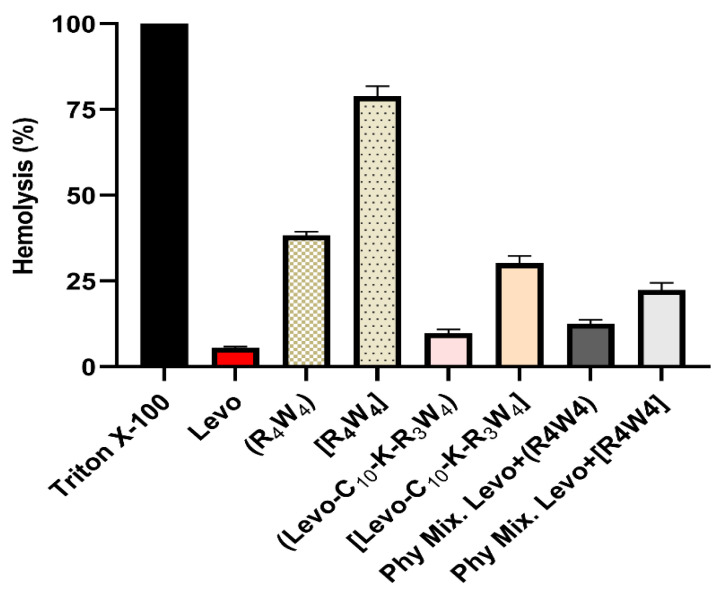
The data represent the percentage hemolysis of the Levo and the peptides (linear and cyclic) alone and their chemical conjugates and physical mixture at 500 µg/mL. The experiment was conducted in triplicate.

We designed an experiment to conduct the hemolytic effect of the Levo, peptides (linear and cyclic), covalent conjugates, and physical mixtures of Levo and peptides. The percentage of hemolysis for each test compound observed at the highest experimental concentration (500 µg/mL) is presented in Figure 2. While cyclic peptide [R_4_W_4_] showed significant hemolytic activity (~80%) moderate hemolysis was observed for linear peptide (~38%). The covalent conjugates of Levo and peptides (Levo-C_10_-KR_3_W_4_) and [Levo-C_10_-KR_3_W_4_]) exhibit low hemolysis as compared to peptide alone. Interestingly, the physical mixture of Levo and peptides (500 µg/mL (1:1; *w*/*w*)) showed negligible toxicity with ~10% and ~22% hemolysis was observed for Levo + (R_4_W_4_) and Levo + [R_4_W_4_], respectively. This significant decrease in hemolytic activity can be attributed to the lowered dose of the cyclic peptide without compromising its antibacterial potency. The results indicate that further exploration of different combinations of antibiotics and the cyclic antibacterial peptides can lead to some breakthrough discovery in antibiotic development against drug-resistant strains. 

### 2.5. Cytotoxicity Assay

**Figure 3:**
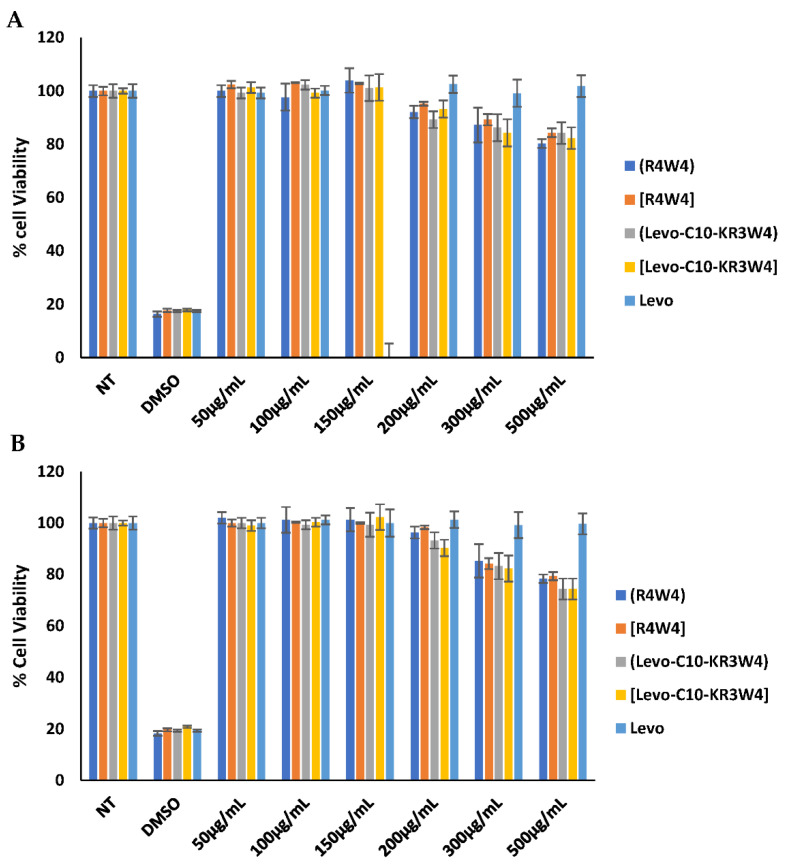
Cytotoxicity of peptides and their conjugates in (**A**) MDA-MB-231 cells and (**B**) in HEK-293 cells after 48 h incubation.

One randomly selected human breast cancer cell line and one normal human kidney cell line were used to evaluate the cytotoxicity of synthesized compounds. Therefore, a cell viability assay was used to perform in vitro cytotoxicity of Levo (R_4_W_4_), [R_4_W_4_] and their conjugates with Levo on triple-negative breast cancer cells (MDA-MB-231) and on normal human embryonic kidney cells (HEK-293) after 48 h incubation. Figure 3 shows that the Levo was non-toxic on both the cell lines up to the tested concentration of 500 µg/mL. The linear peptide (R_4_W_4_) and the cyclic [R_4_W_4_] did not show any cytotoxicity up to 200 µg/mL in both the cell lines. However, they demonstrated ~90% cell viability in MDA-MB-231 at 300 µg/mL, whereas they showed ~85% cell viability at the same concentration as the normal HEK-293 cells. Similar trends were also observed in the cell viability of Levo-conjugates on these cell lines. It concludes that the antimicrobial peptides and their conjugates with Levo are non-toxic up to 200 µg/mL, a much higher concentration than their MIC values.

### 2.6. Time Kill Kinetics

**Figure 4:**
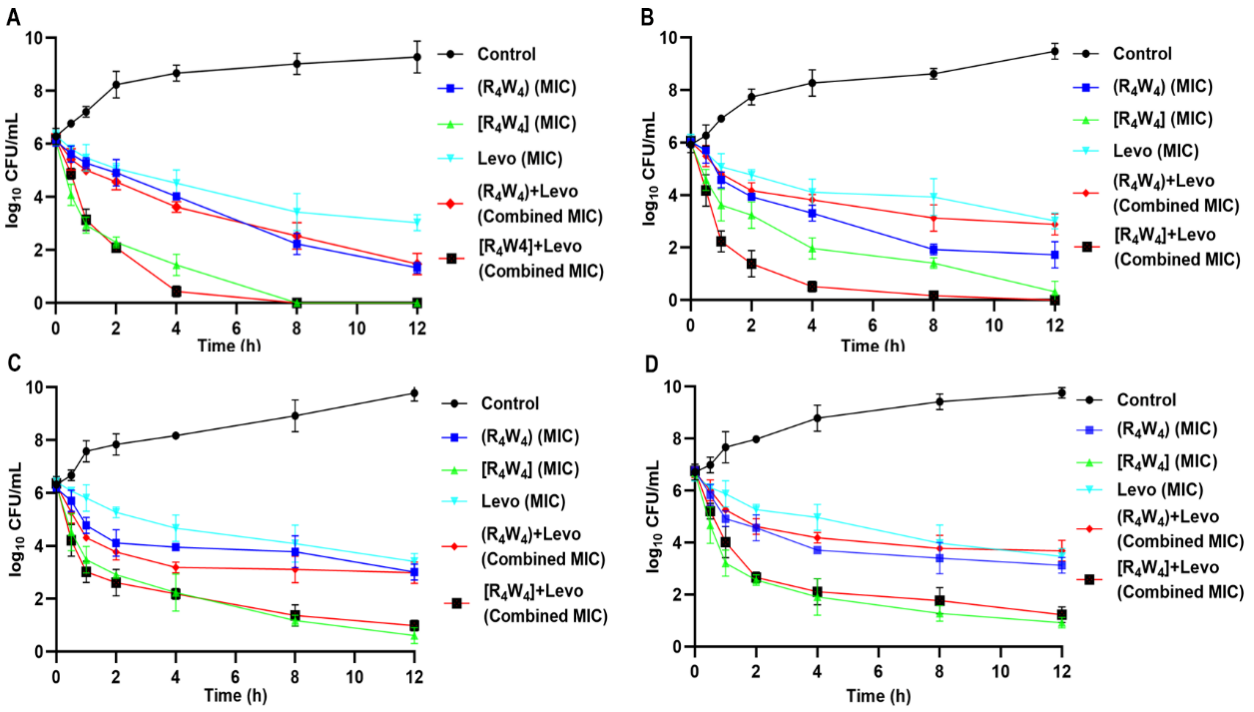
Time-dependent kill study of the test compounds against drug-resistant bacterial strains of (**A**) *S. aureus* (ATCC BAA-1556), (**B**) *E. coli* (ATCC BAA-2452), (**C**) *P. aeruginosa* (ATCC BAA-1744), and (**D**) *K. pneumoniae* (ATCC BAA-1705). The data represent the results of the experiment performed in triplicate.

We conducted a kill kinetic assay of Levo and peptides (linear or cyclic) alone and their physical mixture against drug-resistant bacterial strains of *S. aureus* (ATCC BAA-1556), *E. coli* (ATCC BAA-2452), *P. aeruginosa* (ATCC BAA-1744), and *K. pneumoniae* (ATCC BAA-1705). The purpose of this study was to investigate the time-dependent killing action of the test compounds against antibiotic-resistant strains. Figure 4A reveals that the cyclic [R_4_W_4_] completely eradicated the growth of MRSA in 8 h, as compared to Levo. In contrast, when cyclic [R_4_W_4_] mixed with Levo, it significantly inhibited the growth (~90%) of MRSA in 4 h, pointing to the physical mixture’s effectiveness compared to the peptide or the Levo alone. However, the linear peptide (R_4_W_4_) could not achieve significant growth inhibition even after 12 h, clearly indicating the potency of cyclic peptide over its linear counterpart.

Interestingly, the physical mixture of the linear peptide with Levo could not exert efficient time-dependent killing of the MRSA (Figure 4A). Similar trends were observed for other bacterial strains used in the studies. For instance, in the case of *E. coli*, the cyclic [R_4_W_4_] exhibited a complete inhibition after 12 h. Still, the physical mixture of cyclic peptide [R_4_W_4_] with Levo showed almost complete inhibition within 4 h of exposure, showing the superiority of the physical mixture compared to the peptide or the antibiotic alone (Figure 4B). The linear peptide (R_4_W_4_) could not significantly inhibit the growth of both *P. aeruginosa* (ATCC BAA-1744) and *K. pneumoniae* (ATCC BAA-1705). However, cyclic peptide [R_4_W_4_] exhibited a remarkable growth inhibition activity against both Gram-negative bacterial strains (Figure 4C,D).

Interestingly, there was an apparent time reduction when we compared the cyclic [R_4_W_4_] to the Levo. Overall, the results demonstrated the rapid killing action of cyclic peptide alone and in a physical mixture with Levo (Figure 4). Although, kill kinetics studies are frequently conducted to test the effectiveness of the antibacterial agents. However, to the best of our understanding, this is the first report describing the kill kinetics of the antibiotic with the synthetic cyclic antibacterial peptide [R_4_W_4_]. We can safely speculate from this study that the physical combination of the potent antibiotic with the promising antibacterial peptide molecules can make the drug-resistant strains sensitive.

## 3. Materials and Methods

### 3.1. Chemicals and Reagents

The Fmoc/tBu protected amino acid building blocks, preloaded 2 chlorotrityl resin, and peptide synthesis reagents were purchased from Chem-impex International Inc, (Wood Dale, IL, USA). Solvents and other reagents were purchased from Millipore Sigma (Milwaukee, WI, USA). All the cell culture supplies were purchased from Corning (Christiansburg, VA, USA) and Fisher Scientific (Waltham, MA, USA). The human triple-negative breast cancer cell line (MDA-MB-231, ATCC No. HTB-26), and normal kidney cell line (HEK-293, ATCC No. CRL-1573) were purchased from ATCC (Manassas, VA, USA). CellTiter 96^®^ AQueous One Solution cell proliferation assay (MTS) was purchased from Promega (Madison, WI, USA). Cell culture was carried out at 37 °C with 5% CO_2_ in a Forma incubator using a T-75 flask.

### 3.2. Design and Synthesis of Levo-[W_4_R_3_K] Conjugate

The peptides were synthesized using Fmoc/tBu solid-phase peptide synthesis (SPPS) with protected amino acids building blocks as described in Scheme 1, and the procedure reported previously from our lab [23,30]. In brief, the sequence of linear protected peptide (NH_2_-R(Pbf)-R(Pbf)-R(Pbf)-W(Boc)-W(Boc)-W(Boc)-W(Boc)) was assembled on Trp(Boc)-2-chlorotrityl resin on 0.3 mmol scale. A lysine (K) residue was coupled using Dde-Lys(Fmoc)-OH (4 equiv.), HBTU (4 equiv.), DIPEA (8 equiv.) in the DMF to the peptidyl resin for 2 h, followed by Fmoc deprotection using 20% piperidine in DMF (10 min × 2 times) to provide NH_2_ group. The amino group was coupled with 10-hydroxydecanoic acid (10-HDA, 4 equiv.) using HBTU (4 equiv.) and DIPEA (8 equiv.) in DMF for 3 h. Subsequently, Levo was conjugated via ester bond using a carboxylic group of Levo and the available hydroxyl group on peptidy resin as depicted in Scheme 1. Levo conjugation with the linear peptide (R_4_W_4_) was done according to the conditions described by Ghaffar et al. [31] using HBTU (4.0 eq.)/DIPEA (4.2 eq.), and DMAP (0.1 eq.) on the solid phase. Dde group at N terminus was removed by treating peptidyl resin with 2%hydrazine hydrate in DMF (10 min × 2 times) followed by DMF wash (3 times). After linear peptide containing Levo was assembled on the resin, a mixture of dichloromethane (DCM):2,2,2-trifluoroethanol (TFE): acetic acid (AcOH) (7:2:1 *v*/*v*/*v*, 150 mL) was added to remove the resin, followed by cyclization of side-chain protected peptide overnight under nitrogen using 1,3-diisopropylcarbodiimide (DIC)/1-hydroxy-7-azabenzotriazole (HOAt) (0.3 mmol, 3 equiv.) according to the protocol published by Oh et al. [24]. A test cleavage using a freshly prepared cleavage cocktail of reagent R (90% trifluoroacetic acid (TFA): 2% anisole: 3% thioanisole, and 2–3 mg of dithiothreitol (DTT)) for a small amount of peptidyl resin confirmed the cyclization by MALDI-TOF mass spectrometry. The reaction mixture was evaporated to a minimal amount followed by treatment with reagent R for 6 h and precipitation of peptide using the ice-cold diethyl ether. The precipitated conjugate was purified using RP-HPLC. A gradient of water and acetonitrile (ACN) in 0.1% trifluoroacetic acid (TFA, *v*/*v*) was mobile phase used to purify the peptide on a Shimadzu HPLC system (LC-20AP) (Canby, OR, USA) using a C18 preparatory column (00G-4436-P0-AX Gemini Prep C18, 10 µm particle size, 250 × 21.2 mm, and 110 Å pore size). HPLC fractions showing similar mass were lyophilized to obtain powdered peptides (≥95% purity) used in bioassay. The cyclized peptide [R_4_W_4_] was obtained in ~40% yield and conjugates of Levo with cyclic peptide [Levo-C_10_-KR_3_W_4_] or linear peptides (Levo-C_10_-KR_3_W_4_) were obtained with a lower yield (~20%). MALDI-TOF (*m*/*z*) for synthesized compounds; linear peptide (R_4_W_4_), [C_68_H_90_N_24_O_9_]: calcd, 1386.7323; found, 1388.0632 [M + 2H]^+^; for cyclic peptide [R_4_W_4_], [C_68_H_88_N_24_O_8_]: calcd, 1368.7217; found, 1368.0730 [M]^+^; for linear conjugate (Levo-C_10_-KR_3_W_4_), [C_96_H_128_FN_25_O_14_]: calcd, 1874.0057; found, 1873.9719 [M]^+^; and cyclic conjugate [Levo-C_10_-KR_3_W_4_], [C_96_H_126_FN_25_O_13_]: calcd, 1855.9951; found, 1855.1249 [M]^+^.

### 3.3. Antibacterial Activity

#### 3.3.1. Bacterial Strains

The antibacterial activity of synthesized compounds was determined against a range of susceptible and drug-resistant bacterial strains. The antibacterial activities of all test compounds alone and in combination with Levo were evaluated against the following bacterial strains; *Staphylococcus aureus* (ATCC 29213 and ATCC BAA-1556), *Escherichia coli* (ATCC 25922 and ATCC BAA-2452), *Pseudomonas aeruginosa* (ATCC 27883 and ATCC BAA-1744), and *Klebsiella pneumoniae* (ATCC 13883 and ATCC BAA-1705). All bacterial strains employed in this study were procured from VWR International Inc (Pasadena, CA, USA), and propagated as per the recommendation of the American Type Culture Collection (ATCC).

#### 3.3.2. Determination of Minimal Inhibitory Concentrations (MICs)

Antibacterial susceptibility testing was carried out using a standard microtiter dilution method recommended by clinical and laboratory standard institute (CLSI) and measured as minimum inhibitory concentration (MIC), the lowest peptide concentration that inhibited bacterial growth [35]. Briefly, cells were grown overnight at 37 °C in the broth recommended for each strain and were diluted in cation-adjusted Mueller–Hinton Broth (CAMHB). An aliquot of an overnight culture of bacteria was diluted in 1mL normal saline to achieve 0.5 McFarland turbidity (1.5 × 10^8^ bacterial cell CFU/mL). A total of 60 µL of the 0.5 McFarland solution was added to 8940 µL of MH media (1/150 dilution). An amount of 100 µL MH media was pipetted into the sterile plate wells except for the first row of the plate. An amount of 200 µL of 100 µg/mL test compound was added by pipette into the first row and serially diluted with the MH media sterile 96 wells using a multi-channel pipette except the last row. An amount of 100 µL aliquot of bacteria solution was added to each well, and the plate was incubated at 37 °C for 18–24 h. All experiments were performed in triplicate. 

#### 3.3.3. Checkerboard Assay

The checkerboard method was used to assess the MIC of Levo in combination with antimicrobial peptides. The assay was conducted based on the broth microdilution method in accordance with the CLSI protocols using cation-adjusted Mueller–Hinton broth [36,37]. Levo and peptides were two-fold serially diluted in a horizontal and vertical orientation, respectively, in a 96-well microtiter plate. The assay was performed by taking twice the MICs of Levo and peptides alone determined against various bacterial strains as the highest test concentration. Levo was tested as 11 point, 2-fold serial dilutions across the assay plate (from 1–11) in combination with a 7 point, a 2-fold serial dilution of the peptides down the assay plate. Two-fold serial dilution in row H (from 1–11) for Levo antibiotic alone was performed to determine the MIC value for each test compound. In column 12 (A-G) down the assay plate, 2-fold serial dilution of the peptide alone was performed. Then, 100 µL of test specimen (peptide and Levo alone or a peptide/Levo combination) were inoculated with 100 µL of the bacterial suspension (1 × 10^5^ CFU/mL). MH medium was used as a negative control, and the peptide or Levo alone was used as a positive control. After overnight incubation at 37 °C, the MIC was defined as described above. Synergistic interactions were expressed as the fractional inhibitory concentration index (FICI), which was calculated as follows: FICI = FICa + FICb, where FICa and FICb are the MICs of the peptides in the combination divided by the MICs of the peptides alone and the MICs of the antibiotics in the combination divided by the MICs of the antibiotics alone, respectively. FICI ≤ 0.5, 0.5 < FICI ≤ 1.0, and 1.0 < FICI ≤ 2.0 were defined as synergy, addition, and indifference, respectively [38,39]. The results were collected from 3 independent experiments.

### 3.4. Hemolysis Assay

Hemolytic activity of Levo and peptides alone and in combination was determined using human red blood cells (hRBC) purchased from BioIVT (Hicksville, NY, USA). The hRBC were centrifuged for 15 min to remove the buffy coat and washed 3 times with phosphate buffer saline (100 mM NaCl, 80 mM Na_2_HPO_4_, 20 mM NaH_2_PO_4_, pH 7.4). The assay was conducted in triplicate by mixing 75 µL of peptide solution (2-fold serial dilution) with 75 µL of a 4% (*v*/*v*) hRBC suspension in phosphate buffer saline. The plates were incubated for 2 h at 37 °C without agitation and centrifuged at 1000× *g* for 10 min. Aliquots (100 μL) of the supernatant were transferred to 96-well plates, where hemoglobin release was measured spectrophotometrically at 567 nm. Percent hemolysis was calculated by the following formula:

Percentage hemolysis = 100 × [(A − A_0_)/(At − A_0_)]

where A represents the absorbance of peptide sample at 567 nm and A_0_ and At represent zero percent and 100% hemolysis determined in phosphate buffer saline and 1% Triton X-100, respectively.

### 3.5. Cytotoxicity Assay

The in vitro cytotoxicity of the synthesized peptides, and their conjugates with Levo was determined in the MDA-MB-231 and HEK-293 cell lines. The MDA-MB-231 and HEK-293 cells were cultured in DMEM/F-12 (cat # 11330-032, Corning, VA, USA), 100 IU/mL penicillin, and 100 IU/mL streptomycin supplemented with fetal bovine serum (FBS). Approximately, 10,000 cells per 0.1 mL were seeded in each well in a 96-well plate using a multichannel pipette and were allowed to adhere to the bottom of the plate for 24 h in the incubator. After 24 h, the cells were inspected for their health and confluency. Different concentrations of the peptides and their conjugates were added to each well in triplicate and incubated for 48 h at 37 °C in a humidified atmosphere of 5% CO_2_. After 48 h, 20 μL of the 3-(4,5-dimethylthiazol-2-yl)-5-(3-carboxymethoxyphenyl)-2-(4-sulfophenyl)-2H-tetrazolium (MTS) reagent was added to each well using a multichannel pipette. The 96-well plates were centrifuged at 1000 rpm for 1 min to ensure the settling of the MTS reagent, and after that, the plates were incubated for additional 3 h. 96 well plate was measured for absorbance at 490 nm using SpectraMax M2 microplate spectrophotometer (Molecular Devices, LLC. San Jose, CA, USA) to determine cell viability. Non-Treated (NT) cells served as negative control whereas the DMSO 30% (*v*/*v*) was used as a positive control. The% cell viability was calculated using the following formula;

$$\%\;\mathrm{cell}\;\mathrm{viability}=\frac{\left(\mathrm{OD}\;\mathrm{value}\;\mathrm{of}\;\mathrm{cells}\;\mathrm{treated}\;\mathrm{with}\;\mathrm{compounds}\right)-\left(\mathrm{OD}\;\mathrm{value}\;\mathrm{of}\;\mathrm{culture}\;\mathrm{medium}\right)}{\left(\mathrm{OD}\;\mathrm{value}\;\mathrm{of}\;\mathrm{control}\;\mathrm{cells}\right)-\left(\mathrm{OD}\;\mathrm{value}\;\mathrm{of}\;\mathrm{culture}\;\mathrm{medium}\right)}\times 100$$

### 3.6. Time-Kill Kinetics Assay

The time course of bacterial killing was studied by the exposure of overnight grown cultures of antibiotic-resistant strains of MRSA (ATCC BAA-1556), *E. coli* (ATCC BAA-2452), *P. aeruginosa* (ATCC BAA-1744), and *K. pneumoniae* (ATCC BAA-1705). Levo and peptides alone and in combination at MIC were tested against the above-mentioned bacterial strains. Test tubes containing Mueller–Hinton (MH) broth supplemented with Levo and peptides, alone and in combination, were inoculated with overnight grown bacterial culture (1.5 × 10^8^ CFU/mL) and incubated at 37 °C. Aliquots were sampled at 0, 0.5, 1, 2, 4, 8, and 12 h time points, then diluted and plated on the Muller–Hinton agar plate. After 24 h incubation at 37 °C, CFU count was performed using the standard formula. Untreated bacterial culture was used as a control. Data were obtained from 2 independent experiments performed in triplicate.

## 4. Conclusions

The conjugates of Levo with a potent antibacterial peptide [R_4_W_4_] were tested to provide synergistic activity against selected strains of Gram-positive and Gram-negative bacteria. The covalent conjugates of peptide and Levo were successfully synthesized, characterized, and purified using MALDI-MS and RP-HPLC. The antibacterial activity of the tested compound demonstrated a weaker antibacterial activity for covalent conjugates as compared to combination (physical mixture/noncovalent) and Levo. Therefore, a checkerboard assay was performed, which depicted a synergistic activity between cyclic peptide and Levo. However, it was also worthy of observing that the physical mixture of Levo with peptide, especially cyclic peptide, not only maintained the antibacterial potency against drug-resistant *P. aeruginosa* (ATCC BAA-1744) and *K. pneumoniae* (ATCC BAA-1705) but also significantly improved the antibacterial potency compared to the antibiotic or the peptide alone. Synergistic studies revealed that the cyclic peptide showed remarkable synergy with levofloxacin against all studied strains except for the wild-type strain of *S. aureus* (ATCC 29213), in which the partial synergy was observed. Hemolytic assay results showed that the hemolytic effect of cyclic peptide [R_4_W_4_] significantly reduced in the combination (physical mixture) with Levo without compromising its antibacterial potency. (R_4_W_4_), [R_4_W_4_], and their conjugates with Levo were found not cytotoxic up to 200 µg/mL to the MDA-MB-231 and HEK-293 cells, a much higher concentration than their MIC values. Furthermore, the time-kill kinetic study results also point towards the effectiveness of a combination of the potent antibiotic Levo with cyclic synthetic antimicrobial peptide [R_4_W_4_]. It can be speculated that further exploration of this combination approach can lead to some breakthrough discovery in antibiotic development against drug-resistant strains [40,41]. More studies are needed to translate this approach to the bedside.

## Figures and Tables

**Table 1:** Antibacterial activity of synthesized compounds.

Bacterial Strain	MIC (µg/mL) (µM) ^a^						
	Levo	(R_4_W_4_)	[R_4_W_4_]	(Levo-C_10_-KR_3_W_4_)	[Levo-C_10_-KR_3_W_4_]	Levo + (R_4_W_4_) ^b^	Levo + [R_4_W_4_] ^b^
*S. aureus * (ATCC 29213)	3.13	6.25	3.13	12.5	25	3.13	3.13
	(8.66)	(4.51)	(2.28)	(6.67)	(13.45)		
*S. aureus * (ATCC BAA-1556) ^c^	3.13	6.25	3.13	12.5	50	3.13	6.25
	(8.66)	(4.51)	(2.28)	(6.67)	(26.90)		
*E. coli * (ATCC 25922)	50	25	6.25	50	50	12.5	12.5
	(138.36)	(18.03)	(4.55)	(26.68)	(26.90)		
*E. coli * (ATCC BAA-2452) ^d,e^	50	50	12.5	50	100	25	12.5
	(138.36)	(36.06)	(9.11)	(26.68)	(53.79)		
*P. aeruginosa * (ATCC 27883)	0.78	25	12.5	100	100	1.56	1.56
	(2.16)	(18.03)	(9.11)	(53.36)	(53.79)		
*P. aeruginosa* (ATCC BAA-1744) ^e^	0.78	25	12.5	100	>100	1.56	0.78
	(2.16)	(18.03)	(9.11)	(53.36)	(>53.79)		
*K. pneumoniae* (ATCC 13883)	25	50	12.5	25	50	25	12.5
	(69.18)	(36.06)	(9.11)	(13.34)	(26.90)		
*K. pneumoniae* (ATCC BAA-1705) ^f^	50	25	12.5	50	50	12.5	12.5
	(138.36)	(18.03)	(9.11)	(26.68)	(26.90)		

^a^ Minimum inhibitory concentration (MIC). Values in the parenthesis are MICs in unit of micromolar. ^b^ Physical mixture composed of Levofloxacin: Peptide molar ratio of 3.85:1 (1:1; *w*/*w*). ^c^ Methicillin, ^d^ New Delhi Metallo-beta-lactamase (NDM-1) positive, ^e^ Carbapenem, and ^f^ imipenem resistant bacterial strains.

**Table 2:** Checkerboard assay using a combination of Levo and peptides.

Bacterial Strain	MIC in Combination (µg/mL)		FICI ^a^			
	Levo/(R_4_W_4_)	Levo/[R_4_W_4_]	Levo + (R_4_W_4_)	Interactive Category	Levo + [R_4_W_4_]	Interactive Category
*S. aureus* (ATCC 29213)	1.56/1.56	0.78/1.56	0.748	Partial synergy	0.748	Partial synergy
*S. aureus* (ATCC BAA-1556)	1.56/3.13	0.78/0.78	0.999	Partial synergy	0.498	Synergy
*E. coli * (ATCC 25922)	25/12.5	6.25/1.56	1	Indifference	0.374	Synergy
*E. coli* (ATCC BAA-2452)	25/12.5	3.13/3.13	0.75	Partial synergy	0.313	Synergy
*P. aeruginosa* (ATCC 27883)	0.78/1.56	0.15/1.56	1.062	Indifference	0.317	Synergy
*P. aeruginosa* (ATCC BAA-1744)	0.39/1.56	0.07/1.56	0.562	Partial Synergy	0.214	Synergy
*K. pneumoniae* (ATCC 13883)	25/12.5	6.25/3.13	1.25	Indifference	0.500	Synergy
*K. pneumoniae* (ATCC BAA-1705)	25/12.5	12.5/1.56	1	Indifference	0.374	Synergy

^a^ The Fractional Inhibitory concentration Index (FICI) was calculated according to the equation: FICA + FICB = (MIC Drug A in combination/MIC Drug A alone) + (MIC Drug B in combination/MIC Drug B alone). FICI was interpreted as follows: FICI < 0.5, synergy; 0.5 ≤ FICI < 1, partial synergy; 1 ≤ FICI < 4, additive effect or indifference; 4 ≤ FICI antagonism.

## Data Availability

Not applicable.

## Supplementary Materials

The following supporting information can be downloaded at: https://www.mdpi.com/article/10.3390/antibiotics11030416/s1, which contains MALDI-TOF and analytical HPLC data of synthesized compounds.
