# Supplementary material for: Combination of Amphiphilic Cyclic Peptide [R4W4] and Levofloxacin against Multidrug-Resistant Bacteria
Source: Antibiotics (Basel). 2022 Mar 20;11(3):416. doi: 10.3390/antibiotics11030416 (PMC8944500; doi:10.3390/antibiotics11030416)
Supplement: Supplementary file 1 [file antibiotics-11-00416-s001.zip › antibiotics-1618708-supplementary.pdf]

antibiotics

MDPI

SUPPLEMENTARY INFORMATION

Combination of Amphiphilic Cyclic Peptide [R_4W_4] and Levofloxacin Against Multidrug-Resistant Bacteria

Muhammad Imran Sajid[1,2], Sandeep Lohan[1], Shun Kato[1], Rakesh Kumar Tiwari[1,*]

[1] Center for Targeted Drug Delivery, Department of Biomedical and Pharmaceutical Sciences, Chapman University School of Pharmacy, Harry and Diane Rinker Health Science Campus, Irvine, CA 92618, USA

[2] Faculty of Pharmacy, University of Central Punjab, Lahore 54000, Pakistan

* Corresponding author:
Rakesh Kumar Tiwari, Ph.D.
Chapman University School of Pharmacy
Harry and Diane Rinker Health Science Campus
#263, 9401 Jeronimo Road
Irvine, CA, 92618, USA
tiwari@chapman.edu; Tel: +1-714-516-5483; Fax: +1-714-516-5481

Content

1. MALDI Mass Spectrums **S2-S6**

2. Analytical HPLC Chromatograms **S7-S9**

1. MALDI-TOF mass spectrum of linear peptide (R_4W_4)

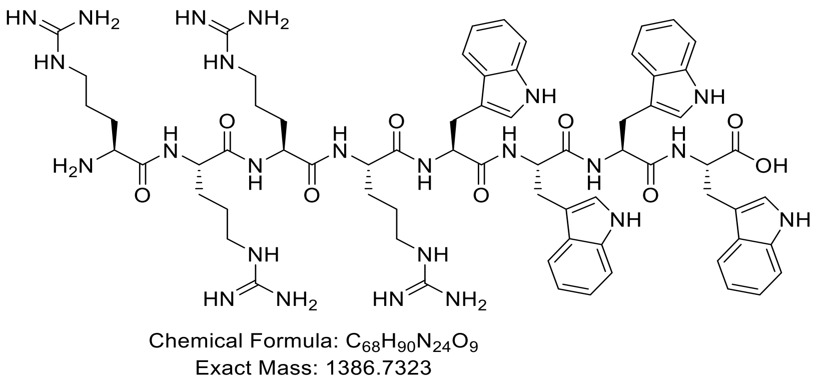

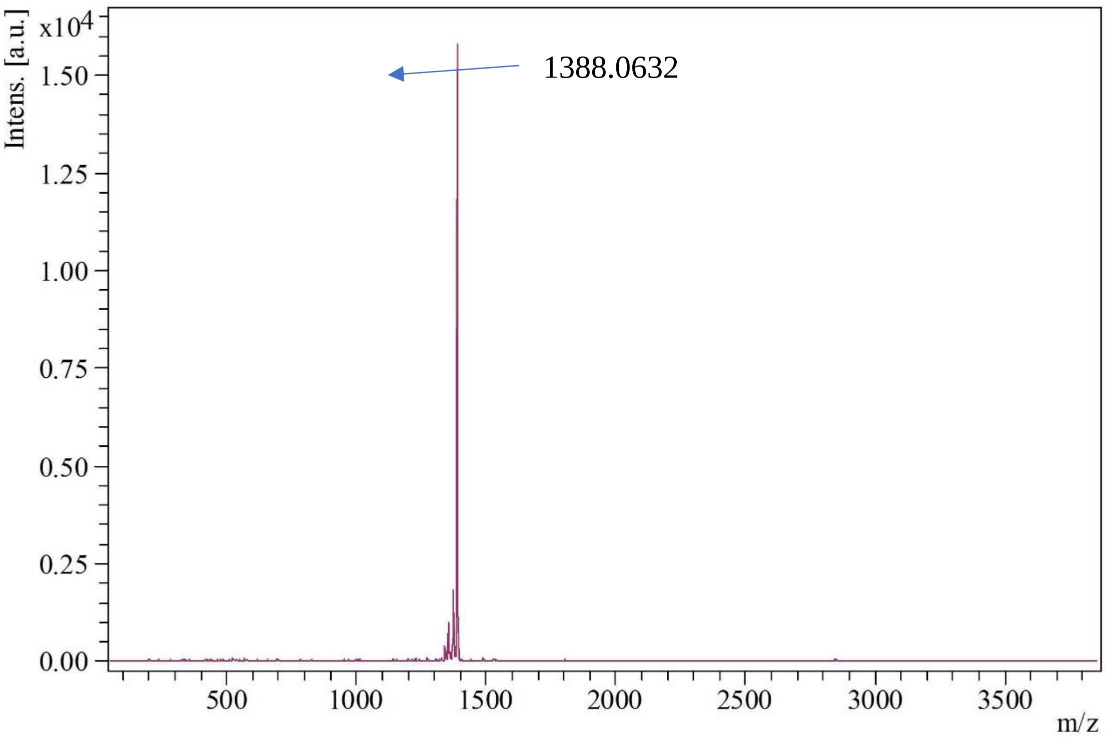

m/z	S/N	Quality Fac.	Res.	Intens.	Area
1340.9943	20	1924	520	207	1363
1354.6184	55	2084	886	733	259
1359.8750	13	2545	762	149	74
1372.0041	13	96.0	538	165	98
1374.4433	95	3748	919	1278	4478
1384.6683	6	4.88	1201	87.1	237
1388.0632	884	235389	957	11904	40730

2. MALDI-TOF mass spectrum of cyclic peptide [R_4W_4]

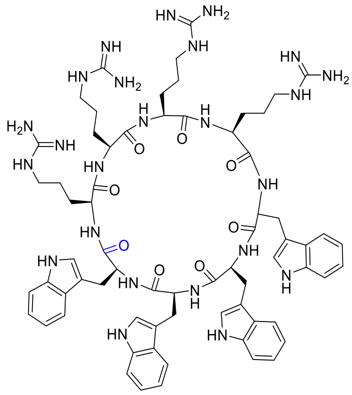

Chemical Formula: $C_{68}H_{88}N_{24}O_8$
Exact Mass: 1368.7217

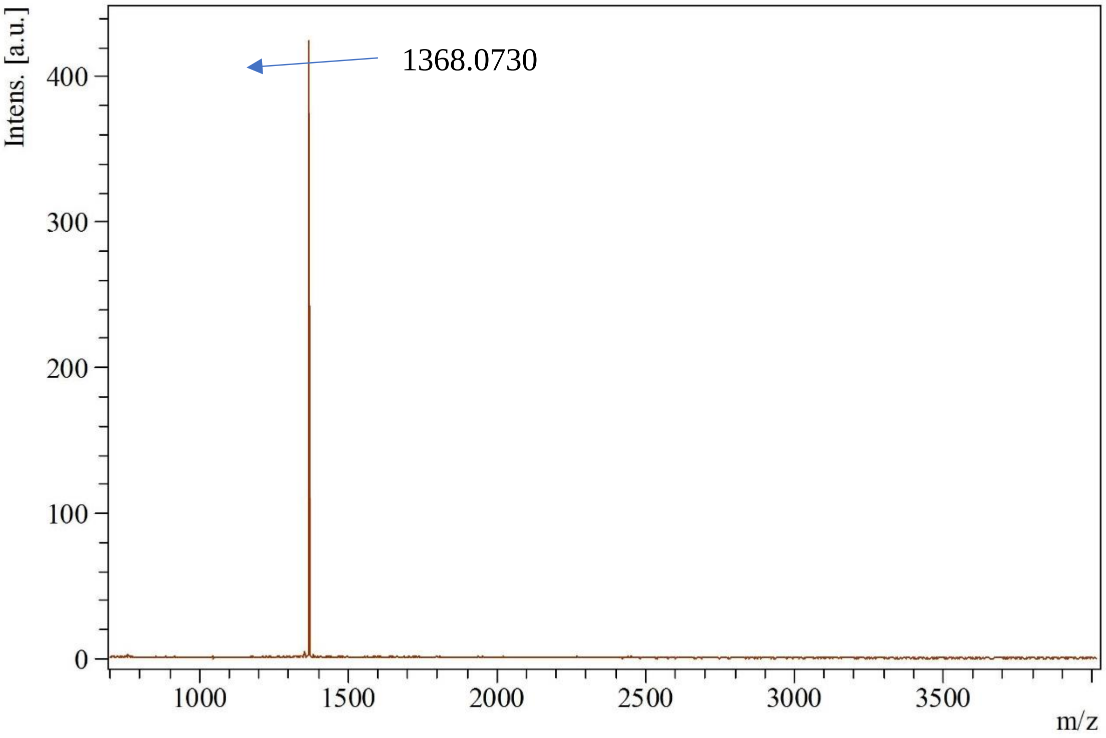

m/z	S/N	Quality Fac.	Res.	Intens.	Area
1368.0730	36	3468	4712	424	288

3. MALDI-TOF mass spectrum of the test cleavage of peptidyl resin to monitor the reaction mixture after 2 h for the coupling of 10-hydroxydecanoic acid (10-HDA) with the linear peptide (Dde-K(NH_2)-(R(Pbf))$_3$(W(Boc))$_4$) on chlorotrityl resin using HBTU and DIPEA in the DMF.

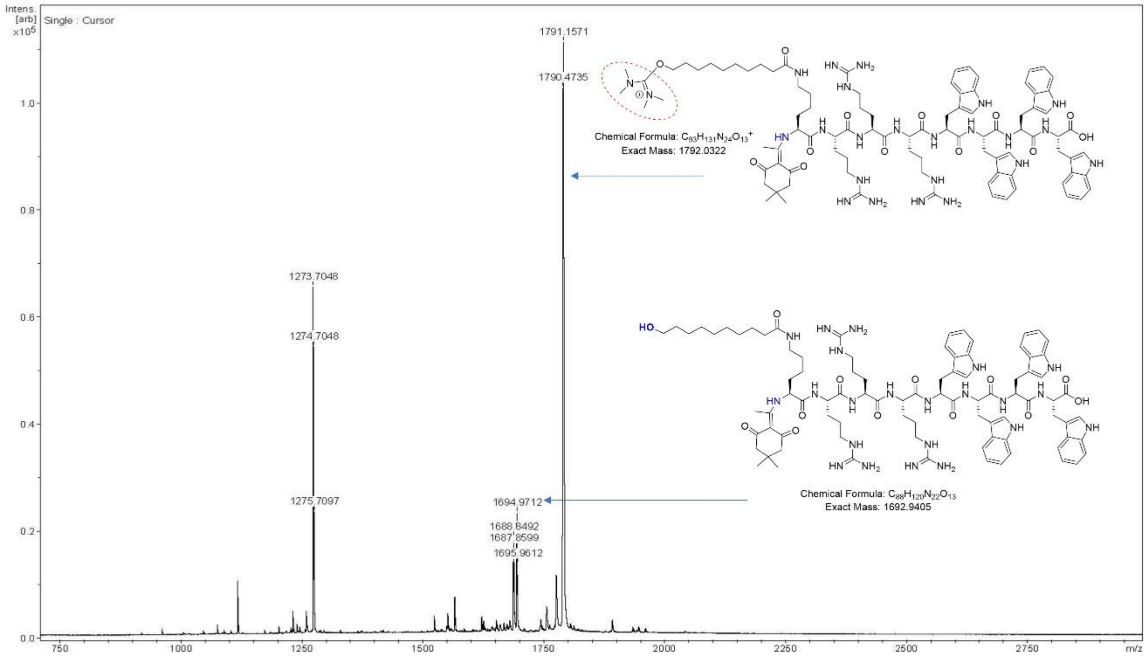

4. MALDI-TOF mass spectrum of linear peptide Levo conjugate (Levo-C_{10}-KR_3W_4)

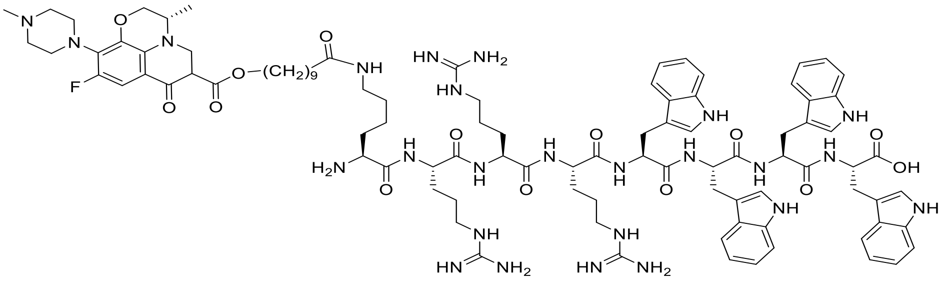

Chemical Formula: $C_{96}H_{128}FN_{25}O_{14}$
Exact Mass: 1874.0057

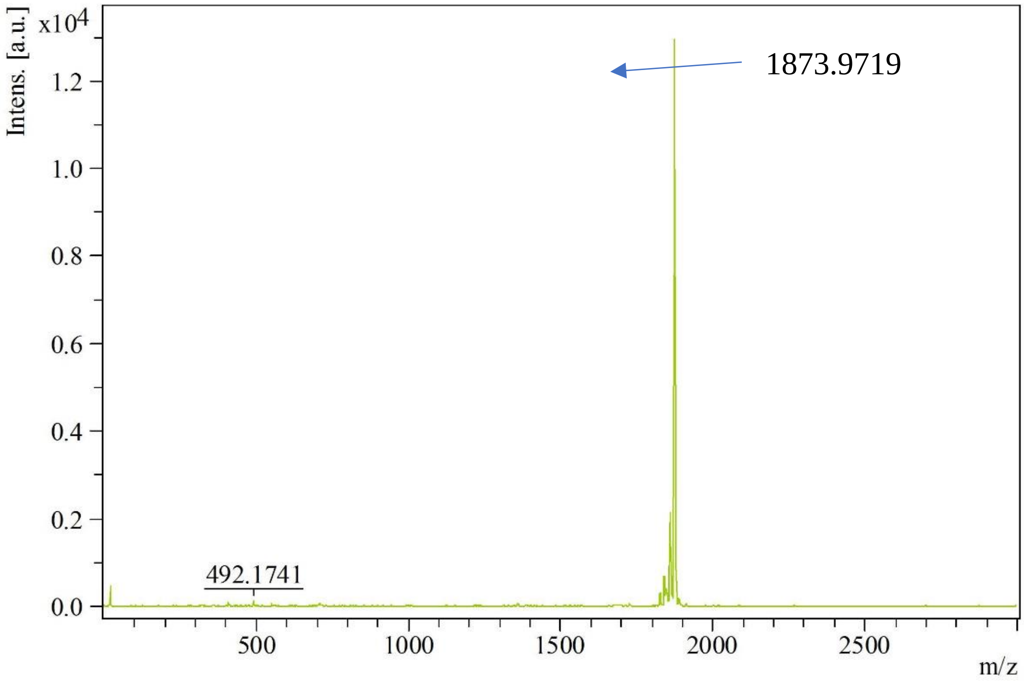

m/z	S/N	Quality Fac.	Res.	Intens.	Area
21.9660	10	158	594	124	5.64
22.0464	6	147	854	81.4	2.25
22.1575	17	700	735	207	6.72
22.2192	19	236	672	237	8.37
22.2936	36	497	705	458	15.4
22.3681	40	464	696	501	17.1
22.4466	22	264	728	278	9.17
22.5222	7	89.6	851	83.3	2.35
22.5953	9	159	581	117	5.46
22.7311	13	166	655	162	7.03
22.8040	7	137	883	92.4	3.02
22.8809	6	207	778	78.9	2.52
23.0239	9	505	582	115	4.84
492.1741	8	1520	1498	108	55.4
1826.6876	15	17117	675	132	1152
1840.1408	41	9663	842	364	2600
1845.8078	21	4278	613	166	1748
1859.8388	117	15364	747	998	8321
1868.1896	9	151	492	88.5	1057
1873.9719	723	2.174316e6	1017	7393	42699

5. MALDI-TOF mass spectrum of cyclic peptide Levo conjugate [Levo-C_{10}-KR_3W_4]

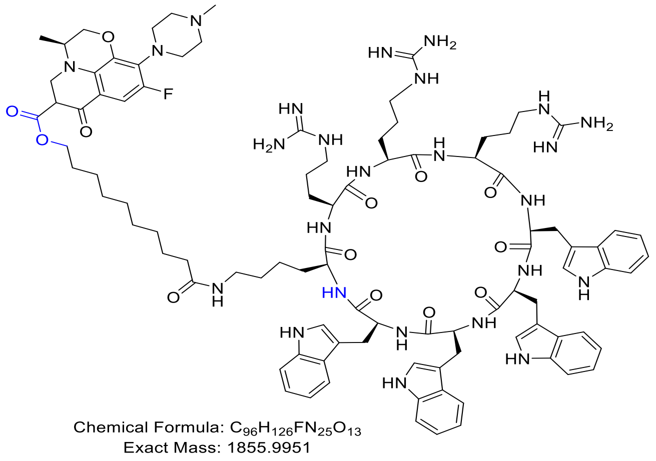

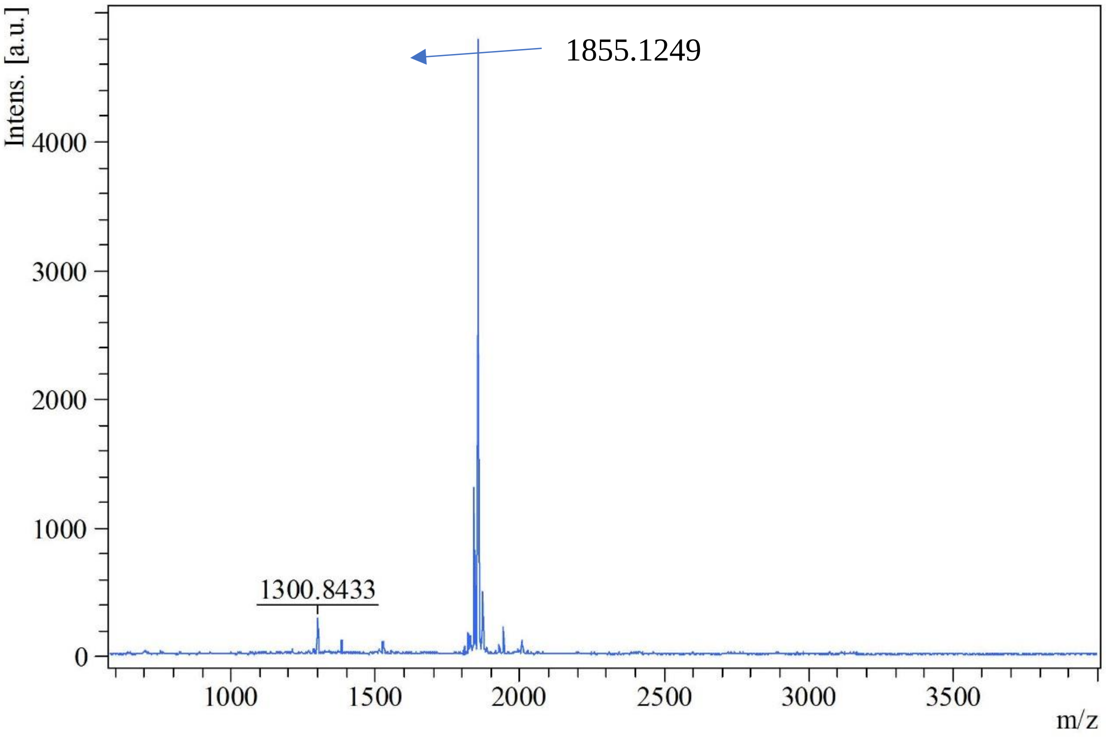

m/z	S/N	Quality Fac.	Res.	Intens.	Area
1300.8433	16	58348	2503	292	390
1821.3029	8	3412	1982	130	422
1841.0730	63	168137	2404	1052	2808
1855.1249	236	225664	2436	3910	10399
1868.0990	8	454	2405	129	372
1871.1541	23	3250	2597	437	984
1942.1810	11	32376	3289	191	387
2007.1052	6	4492	2919	93.8	236

6. Analytical HPLC analysis. The purity of linear (R_4W_4) and cyclic [R_4W_4] peptides and their respective Levo conjugates were determined using a reverse-phase analytical HPLC (Shimadzu; LC-20ADXR). Method 1 was used to determine the purity of the linear (R_4W_4) and cyclic [R_4W_4] peptides. Method 2 was used to determine the purity of Levo conjugates of linear and cyclic peptides.

Method 1:

Column: Phenomenex (Luna), 4 µm, C18, 150 × 4.6 mm
Flow rate: 0.5 ml/min
Mobile Phase: Buffer A-Water containing 0.1% TFA
Buffer B-Acetonitrile containing 0.1% TFA
Gradient

Time (min)	Buffer B (%)
0.01	5
2	5
5	10
25	70
27	95
32	95
32.01	5
38	5

Method 2

Column: Phenomenex (Luna), 4 µm, C18, 150 × 4.6 mm
Flow rate: 0.5 ml/min
Mobile Phase: Buffer A- Water containing 0.1%TFA
Buffer B- Methanol containing 0.1%TFA

Time (min)	Buffer B (%)
0.01	5
2	5
10	20
25	65
27	95
32	95
32.01	5
38	5

6A. HPLC Chromatograms of linear peptide (R_4W_4)

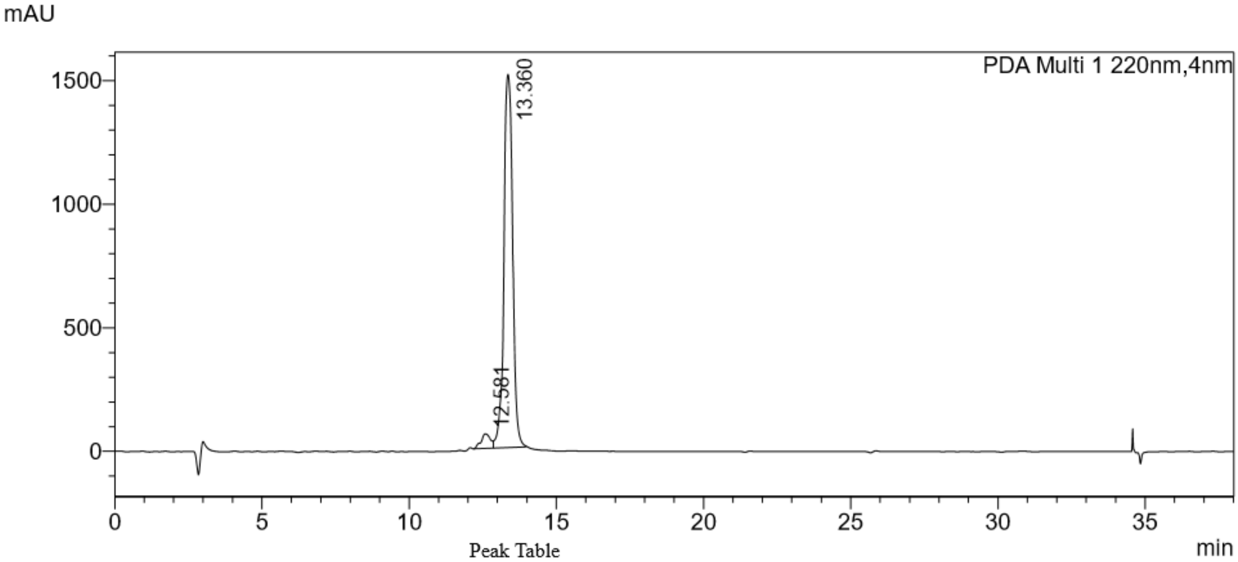

Peak Table

PDA Ch1 220nm

Peak#	Ret. Time	Area	Height	Area%
1	12.581	1338545	59166	4.122
2	13.360	31133436	1509567	95.878
Total		32471981	1568733	100.000

6B. HPLC Chromatograms of cyclic peptide [R_4W_4]

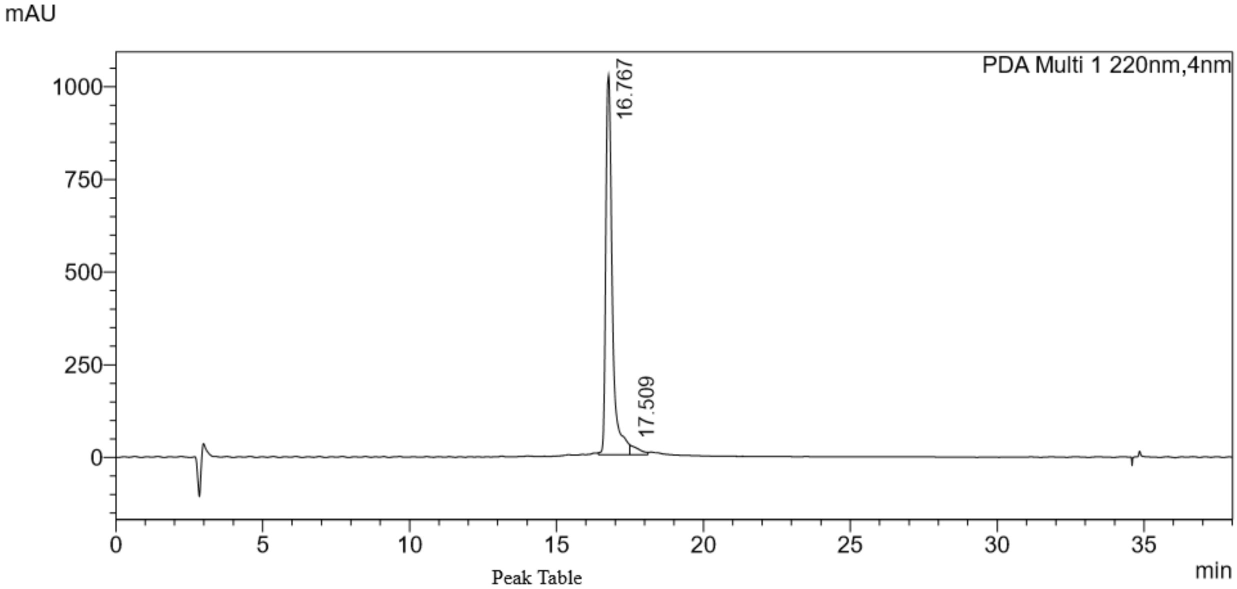

Peak Table

PDA Ch1 220nm

Peak#	Ret. Time	Area	Height	Area%
1	16.767	15393017	1022848	96.619
2	17.509	538702	25641	3.381
Total		15931719	1048489	100.000

6C. HPLC Chromatograms of linear peptide levofloxacin conjugate (Levo-C_{10}-KR_3W_4)

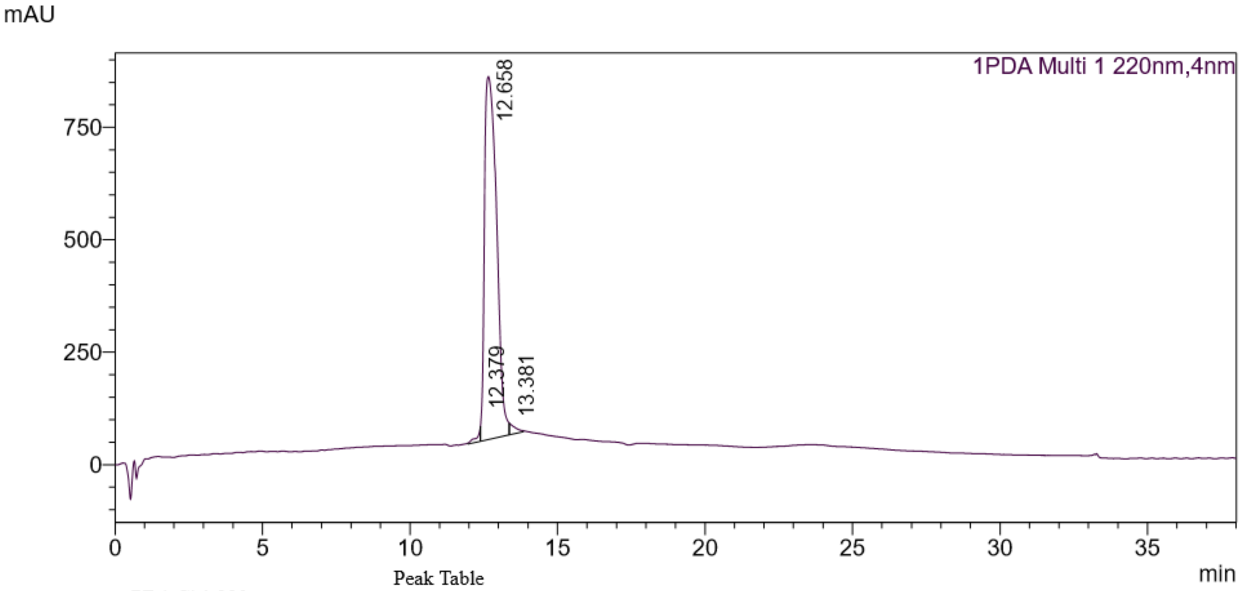

PDA Ch1 220nm

Peak#	Ret. Time	Area	Height	Area%
1	12.379	253107	27989	1.074
2	12.658	22986000	806374	97.512
3	13.381	333426	25126	1.414
Total		23572533	859489	100.000

6D. HPLC Chromatograms of cyclic peptide Levo conjugate [Levo-C_{10}-KR_3W_4]

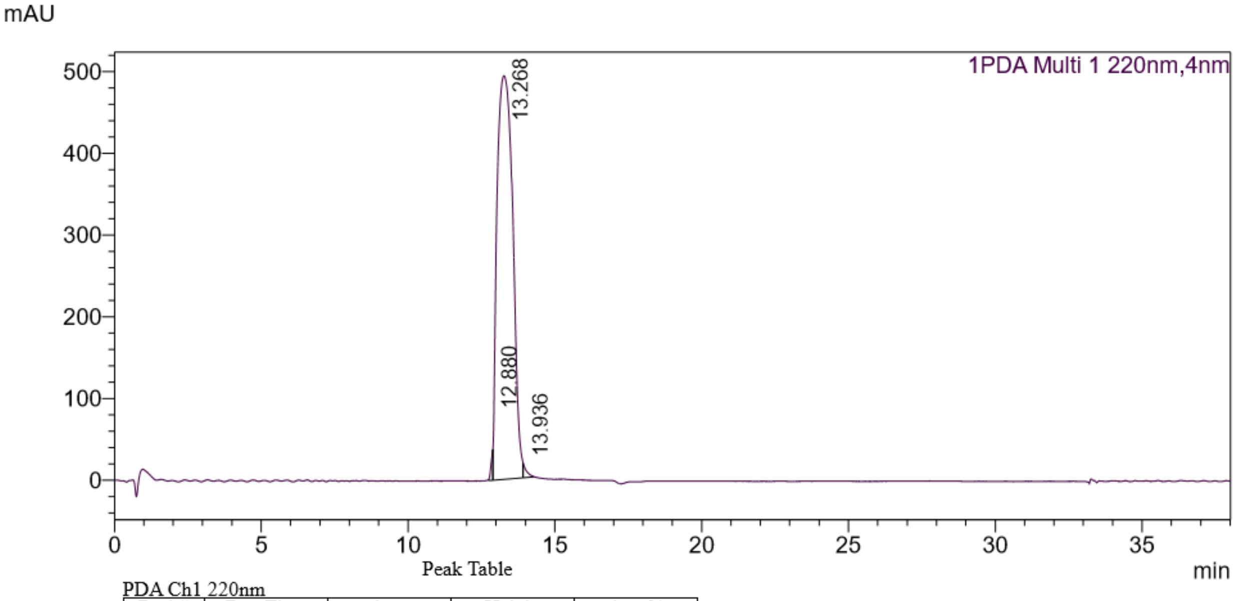

PDA Ch1 220nm

Peak#	Ret. Time	Area	Height	Area%
1	12.880	144586	33350	0.772
2	13.268	18457165	493833	98.543
3	13.936	128313	16528	0.685
Total		18730064	543711	100.000
